# Acute Rheumatic Fever or Post-streptococcal Reactive Arthritis: Two Different Entities

**DOI:** 10.7759/cureus.72687

**Published:** 2024-10-30

**Authors:** Ricardo Silva Veiga, Marta Marques, José Fonseca, Hugo Ventura, Joana Silva Marques

**Affiliations:** 1 Internal Medicine, Hospital São Teotónio, Viseu, PRT; 2 Infectious Diseases, Hospital São Teotónio, Viseu, PRT

**Keywords:** adult onset acute rheumatic fever, group a streptococcal infections, jones criteria, post-streptococcal reactive arthritis, reactive arthritis

## Abstract

The authors present the case of a 71-year-old man with fever, polyarthralgia, and migratory arthritis after the diagnosis of pharyngitis one week earlier. The patient showed unequivocal evidence of recent group A streptococcal (GAS) infection with a significantly elevated antistreptolysin O (ASO) titer that normalized during follow-up. The symptoms, laboratory results, and clinical examination raised uncertainty about whether the patient was suffering from acute rheumatic fever (ARF) or post-streptococcal reactive arthritis (PSReA), as the patient exhibited characteristics of both conditions. These two conditions share a similar origin and, in some cases, lack clear distinction, suggesting that they may be part of the same disease spectrum. The case emphasizes the importance of considering ARF in patients with suspected recent streptococcal infections who present with polyarthralgia and fever, ensuring early and appropriate treatment to prevent complications.

## Introduction

Acute rheumatic fever (ARF) is an acute, non-suppurative, inflammatory complication following group A streptococcus (GAS) infection. While the typical latency period between the GAS infection and the onset of ARF is usually longer (10-28 days), cases have been reported where symptoms began just one week after the initial pharyngitis [1]. With the evolution of healthcare systems and improved access to medical care, the incidence of ARF has significantly decreased in recent decades, especially in developed countries. However, it remains a cause of periodic outbreaks in developing countries due to poor health conditions and specific climatic factors, such as low temperatures and high humidity, which favor streptococcal infections [2].

Reports of ARF in adults are rare, with only a few cases documented in the literature [3-6], as ARF typically affects younger populations. When ARF does occur in adults, it presents a diagnostic challenge, particularly in differentiating between a new Streptococcus infection leading to ARF and a recurrence of the disease initially acquired during childhood [5,7]. Moreover, distinguishing ARF from post-streptococcal reactive arthritis (PSReA) is crucial, as these two conditions share a common etiology but present different clinical patterns and timelines [8]. In PSReA, the latency period between the streptococcal infection and the onset of arthritis is usually 10 days or less, compared to more than 21 days in ARF. Additionally, the clinical course of ARF tends to be shorter, typically less than five days, whereas PSReA can last for an average of two months. A notable distinction between the two conditions is the excellent response of ARF to treatment with non-steroidal anti-inflammatory drugs (NSAIDs).

## Case presentation

This case describes a 71-year-old man with a history of hemorrhagic stroke (without sequelae), hypertension, and penicillin allergy. He was on chronic medication with esomeprazole and rosuvastatin. The patient presented to the emergency department reporting a fever of 38.6ºC and joint pain, initially affecting his right ankle and later his left knee. A week prior, he had visited the emergency department with fever and sore throat, for which he was discharged with azithromycin for three days, leading to symptom resolution. Additionally, he reported being vaccinated with the COVID-19 vaccine two weeks before the onset of pharyngitis.

At the time of admission, the patient presented with fever (maximum temperature recorded at 38.3ºC), along with pain, swelling, and erythema in the left knee and mild residual edema in the right ankle. After an orthopedic evaluation, the patient was submitted to left knee arthrocentesis (Table 1), yielding thick citrine fluid with less than 40,000 leukocytes/mm³ and negative microbiological results, ruling out septic arthritis. Excluding this diagnosis in the investigation of acute rheumatic fever is crucial because septic arthritis requires immediate antibiotic treatment to prevent severe complications, such as joint destruction and systemic infection.

**Table 1:** Arthrocentesis results.

Test	First admission	Reference range
Glucose (mg/dL)	82	60-90
Proteins (g/L)	53	11-22
Leukocytes (mm^3^)	<40,000	<200,000
Microcrystals	Negative	

Upon admission, the patient was hemodynamically stable, with an unremarkable oropharyngeal exam and no cardiac murmurs. Other aspects of his physical examination were also normal. Complementary exams, including abdominal ultrasound, chest X-ray, and knee X-ray, showed no significant findings. Laboratory results revealed normocytic and normochromic anemia (hemoglobin: 12.6 g/dL, mean corpuscular volume: 88.7 fL, mean corpuscular hemoglobin: 29.6 pg) and elevated markers of inflammation, including the erythrocyte sedimentation rate and C-reactive protein (Table 2). Respiratory, urinary, and abdominal infections were ruled out. The patient had no personal history of insect bites or animal contact, nor a family history of arthritis, uveitis, skin lesions, or any autoimmune diseases.

**Table 2:** Laboratory study results.

Test	First admission	Day of discharge, results from 12/12/2023	Outpatient re-evaluation results from 8/5/24	Reference range
Leukocytes (U/L)	12.24 x 10^9^	12.17 x 10^9^	8.03 x 10^9^	4.50-11.50
Hb (g/dL)	12.6	12.0	14.8	14.0-18.0
Mean corpuscular volume (fL)	88.7	88.3	89.8	80-95
Mean corpuscular hemoglobin concentration (pg)	29.6	33.8	33	23-32
Erythrocyte sedimentation rate (ESR) (mm)	73			0-10
C-reactive protein (mg/dL)	26.19	3.14	0.45	0.0-0.5
Antistreptolysin O titer (ASO) (IU/ml)	1366		160.8	
Ferritin (ng/dL)	438			30-340
Cyclic citrullinated peptide (U/ml)	0.5			0-10
Anti-nuclear antibodies (IFI)	Negative			
Extractable nuclear antigen (ENA) screen	Negative			
Human leukocyte antigen B27 (HLA-B27)	Negative			
Borrelia burgdorferi serology	Negative			

He was admitted for further evaluation of oligoarthritis and fever. The inflammation in his right ankle and left knee subsided quickly and migrated to his right wrist. Initial antibiotic therapy was withheld, and due to a suspicion of reactive arthritis, the patient was treated with naproxen 500 milligrams twice daily, which led to an improvement in his symptoms. Further tests ruled out autoimmune diseases, and blood cultures were negative, though his antistreptolysin O (ASO) titer was markedly elevated (Table 2). The patient met the modified Jones criteria for acute rheumatic fever (ARF). As a result, due to his penicillin allergy, azithromycin was initiated. A transthoracic echocardiogram was performed to rule out cardiac involvement, showing good biventricular systolic function and no valvular abnormalities suggestive of cardiovascular involvement.

The patient’s condition improved throughout his hospital stay, with a resolution of fever and a reduction in C-reactive protein levels following treatment. Despite this improvement, he continued to experience some inflammation and functional impairment in his right wrist. He was discharged with instructions to continue NSAID therapy and was scheduled for outpatient follow-up. Additionally, his household contacts were also treated with azithromycin to prevent the potential spread of streptococcal infection. However, the NSAID had to be discontinued due to the development of gastric antral vascular ectasia. Later, lab results showed normal ASO titers. Eight months post-discharge, the patient did not present signs of arthritis, although he still experienced mild functional impairment in his right wrist. He remained asymptomatic throughout a 10-month follow-up period.

## Discussion

Our patient presented with fever, migratory oligoarthritis, markedly elevated inflammatory parameters (C-reactive protein and erythrocyte sedimentation rate), and a history of recent pharyngitis. The markedly elevated ASO titer, along with its resolution during outpatient follow-up, strongly supports the diagnosis of a prior GAS pharyngitis. Although ARF presents great variability in its presentation (especially in high-risk populations) [9], a differential diagnosis considered was PSReA, which usually overlaps with ARF. In contrast to ARF, which typically manifests 10-28 days after the initial GAS infection, post-streptococcal reactive arthritis (PSReA) generally begins seven to 10 days following the infection. In our patient's case, the time elapsed until the onset of arthralgias and fever was relatively short, approximately one week, which aligns more with the typical timeline for PSReA. Arthritis of PSReA is cumulative, persistent, and affects small joints and the axial skeleton, with an average duration of two months [10,11]. Although the duration of the right wrist arthritis was more compatible with PSReA than with ARF (in the latter, there is a rapid resolution of symptoms), the arthritis was migratory and affected great joints, which is characteristic of ARF. There is also increased expression of the human leukocyte antigen HLA-B27 allele in PSReA but not ARF. This patient tested negative for HLA-B27, which again contradicts his diagnosis of PSReA [8]. According to the modified Jones criteria [2,9,12] for the diagnosis of ARF, as presented in Table 3, the patient meets one major criterion (arthritis) and two minor criteria (C-reactive protein or erythrocyte sedimentation rate and fever). Although he had concomitant polyarthralgia, joint manifestations can only be counted once as either a major or minor criterion. Assuming that Portugal has a low-risk population, the fever criterion is only positive if the body temperature is equal to or greater than 38.5ºC, which was reported by the patient prior to the admission. During hospitalization, the maximum measured temperature was 38.3ºC.

**Table 3:** Revision of the Jones criteria for the diagnosis of acute rheumatic. ARF: acute rheumatic fever; CRP: C-reactive protein; ESR: erythrocyte sedimentation rate; and GAS: group A streptococcal infection. Adapted from Reference [12].

Diagnosis: initial ARF	Two major manifestations or one major plus two minor manifestations
Diagnosis: recurrent ARF	Two major or one major and two minor or three minor
A. Major criteria	
Low-risk populations	Moderate- and high-risk populations
Carditis (clinical and/or subclinical)	Carditis (clinical and/or subclinical)
Arthritis (polyarthritis only)	Arthritis (monoarthritis, polyarthritis, or polyarthralgia)
Chorea	Chorea
Erythema marginatum	Erythema marginatum
Subcutaneous nodules	Subcutaneous nodules
B. Minor criteria	
Low-risk populations	Moderate- and high-risk populations
Polyarthralgia	Monoarthralgia
Fever (≥38.5°C)	Fever (≥38°C)
ESR ≥60 mm in the first hour and/or CRP ≥3.0 mg/dL	ESR ≥30 mm/h and/or CRP ≥3.0 mg/dL
Prolonged PR interval, after accounting for age variability (unless carditis is a major criterion)	Prolonged PR interval, after accounting for age variability (unless carditis is a major criterion)

In the present case, the clinical team initially considered the hypothesis of reactive arthritis after vaccination against SARS-CoV-2, given the recent history of vaccination (approximately three weeks prior). However, in cases described in the literature of reactive arthritis following COVID-19 vaccination [13], it typically affects the large joints of the lower limbs. It most often occurs within the first week after the administration of the dose. Also, the patient had a negative polymerase chain reaction test for COVID-19 at admission.

Considering the already mentioned continuous spectrum between PSReA and ARF, it is expected that some characteristics of these entities are mixed. However, it is worth noting that what is relevant for the diagnosis of ARF is compliance with the modified Jones criteria, as occurred with our patient.

Other clinical manifestations of ARF include chorea, erythema marginatum, and subcutaneous nodules (the latter being more associated with cardiac involvement). Analytically, patients frequently present leukocytosis with neutrophilia, anemia, and elevated inflammatory parameters. After the introduction of non-steroidal anti-inflammatory drugs, the patient showed clinical improvement and resolution of fever. Despite the absence of alterations in complementary exams or clinical signs suggestive of cardiovascular involvement, the patient still underwent an echocardiogram that revealed no abnormalities suggestive of carditis. The treatment of ARF involves the eradication of the streptococcal infection (usually with penicillin G or macrolides in case of allergy) and treating the respective complications: for arthritis, treatment involves non-steroidal anti-inflammatory drugs until improvement of the inflammatory process that can accelerate recovery and prevent arthritis migration but may delay clinical diagnosis; for carditis, treatment consists of prednisolone (1-2 mg/kg/day) for two to three weeks until clinical improvement and reduction of inflammatory markers [2]. Although it reduces the future risk of streptococcal infection, antibiotic prophylaxis does not affect the course of the arthritis [14].

## Conclusions

ARF and PSReA should be part of the differential diagnosis for adults presenting with acute arthritis in one or more joints. Distinguishing PSReA from ARF can be challenging because both entities have similar symptoms and signs, but the diseases differ in prognosis. Arthritis is the most frequently observed manifestation of ARF, typically involving large joints (knee, elbow, ankle, and wrist). It usually appears three weeks after streptococcal infection and is typically migratory, with complete resolution after about three weeks.

A high index of suspicion is required in patients with a recent history of pharyngitis and symptoms such as arthralgia, fever, chorea, new murmurs on auscultation, or cardiovascular changes, particularly given the current rarity of ARF in adults and developed countries. In this case, the patient fulfilled the modified Jones criteria for ARF. However, the long-term follow-up ultimately allows for a clearer distinction between ARF and PSReA.
